# The influence of high-efficiency particulate air filtration on mortality among multiple myeloma patients receiving autologous stem cell transplantation

**DOI:** 10.1038/s41598-021-91135-0

**Published:** 2021-06-03

**Authors:** Chun-Kuang Tsai, Chiu-Mei Yeh, Ying-Chung Hong, Po-Min Chen, Jin-Hwang Liu, Jyh-Pyng Gau, Chia-Jen Liu

**Affiliations:** 1 Division of Hematology and Oncology, Department of Medicine, Taipei Veterans General Hospital, No. 201 Shipai Road, Sec. 2, Taipei, 11217 Taiwan; 2 Institute of Public Health, National Yang-Ming University, Taipei, Taiwan; 3 Division of Hematology and Oncology, Kaohsiung Veterans General Hospital, Kaohsiung, Taiwan; 4 School of Medicine, National Yang-Ming University, Taipei, Taiwan; 5 Division of Hematology and Oncology, Cheng Hsin General Hospital, Taipei, Taiwan; 6 Chong Hin Loon Cancer and Biotherapy Research Center, and Institute of Biopharmaceutical Sciences, National Yang-Ming University, Taipei, Taiwan

**Keywords:** Myeloma, Health care economics, Myeloma, Haematopoietic stem cells

## Abstract

Autologous stem cell transplantation (ASCT) continues to be the standard treatment for transplant-eligible multiple myeloma (MM) patients. A portion of MM patients received ASCT in an isolation room with high-efficiency particulate air (HEPA) filtration. The effectiveness of the HEPA filtration on reducing treatment-related mortality (TRM) is controversial. We enrolled patients with newly diagnosed MM in Taiwan between 2000 and 2017. The primary endpoint of the study was TRM, which was defined as death within 100 days after ASCT. A total of 961 MM patients received ASCT. Of them, 480 patients (49.9%) received ASCT in an isolation room with HEPA filtration (HEPA group). The median overall survival from ASCT was 7.52 years for the HEPA group and 5.88 years for the remaining patients (non-HEPA group) (*p* = 0.370). The 100-day mortality rate was 1.5% and 1.0% for the HEPA and non-HEPA groups, respectively. In the multivariate analysis, the 100-day mortality had no difference between the HEPA and non-HEPA groups (adjusted hazard ratio 1.65, 95% CI 0.52–5.23). The median cost for ASCT inpatient care was $13,777.6 and $6527.6 for the HEPA and non-HEPA groups, respectively (*p* < 0.001). Although half of MM patients in Taiwan received ASCT in HEPA room, it didn’t affect 100-day mortality.

## Introduction

Multiple myeloma (MM) is a hematologic neoplasm characterized by the clonal proliferation of plasma cells^1^. In the United States, the estimated new MM cases in 2019 were 32,110, which represented 1.6% of all cancers, and MM was the second most common hematologic malignancy^2^. The standardized incidence of MM was 17.0 per 10,000 person-years in Taiwan in 2016. MM therapy has remarkably changed in past decades with the introduction of novel agents^3^. The early mortality rate has been substantially reduced, and the survival rate has doubled^4^.

High-dose chemotherapy with autologous stem cell transplantation (ASCT) prolonged progression-free survival (PFS) and overall survival (OS) in newly diagnosed MM patients who were eligible for transplantation^5–7^. MM patients who received high-dose chemotherapy plus ASCT were often hospitalized in an isolation room with high-efficiency particulate air (HEPA) filtration^8,9^. Krüger et al. presented the results of a multi-center survey to the members of the European Group for Bone and Marrow Transplantation (EBMT) in 1999 and reported that 47.2% of the patients received ASCT in a special ward with HEPA filtration^10^. Another EBMT survey in 2008 revealed that HEPA-filtered rooms were used in 53% of ASCT conditioned without total body irradiation^11^. The latest survey conducted between 2014 and 2015 showed that the use of HEPA-filtered rooms was 68% for ASCT recipients.

The need for environmental HEPA filtration for patients receiving ASCT has not been established. IDSA guidelines suggest considering the use of HEPA-filtered rooms for ASCT recipients who develop prolonged neutropenia, which is the major risk factor of nosocomial aspergillosis^12^. Conversely, rapid engraftment with peripheral blood stem cells and the improvement of supportive care have made ASCT very safe, with a low treatment mortality rate^13,14^. Taiwan is located in southeastern Asia, with a warm humid climate, so ASCT patients in Taiwan might have a high rate of nosocomial infection^15^. Half of the MM patients in Taiwan received ASCT in an isolation room with HEPA filtration, although the efficacy of HEPA filtration for those patients has not been established. Therefore, we conducted a nationwide population-based study to evaluate the benefits and cost of the use of HEPA filtration for MM patients receiving ASCT.

## Patients and methods

### Study population

We used data from the Taiwan Cancer Registry, Cause of Death Data, and National Health Insurance Research Database (NHIRD). Data retrieval and analysis were carried out in the Health and Welfare Data Science Center (HWDC). Taiwan’s NHIRD provides nationwide population-based data for health research. All patients with severe diseases, of which cancers are included, are enrolled in the Registry for Catastrophic Illness Patients (RCIP) and receive copayment exemption under the National Health Insurance (NHI) program. The integration of multiple NHI databases, including RCIP, NHI enrollment files, inpatient and outpatient databases, provides comprehensive information on NHI enrollment and utilization of healthcare resources, including examinations and treatment^16^. Cancer stages and treatment plans are available in the Taiwan Cancer Registry. All the patients’ identification has been encrypted and can be analyzed only in the HWDC. This study has been approved by the Institutional Review Board of Taipei Veterans General Hospital (no. 2020-02-019AC). All methods for the study were performed in accordance with relevant guidelines and regulations of Taipei Veterans General Hospital in Taiwan. The institutional ethical committee waived the informed consent form.

### Study cohort and study design

We enrolled patients with newly diagnosed MM in Taiwan between January 1, 2000 and December 31, 2017 from diagnosis codes according to the International Classification of Diseases, 9th revision, and Clinical Modification (ICD-9-CM) codes (203, 203.0X, and 203.1X) and 10th revision (ICD-10-CM) codes (C90.X). The diagnosis of MM had to be further verified by the RCIP, in which diagnosis was confirmed by pathologic reports. Patients under age 20, without ASCT, or receiving non-melphalan conditioning regimens were excluded. We identified MM patients who received ASCT in an isolation room with HEPA filtration as the HEPA group. Comparatively, those who received ASCT in a ward without HEPA filtration were identified as the non-HEPA group.

### Endpoints

The primary endpoint of the study was treatment-related mortality, which was defined as death within 100 days following receiving ASCT, of which only the first ASCT was analyzed^17^. We used the National Cause of Death Data to identify the date and cause of death. The secondary endpoints included OS, medical expenditures within 100 days, length of stay in hospitals for the treatment course of ASCT, and emergency room visits and readmission within 14 days after discharge. Medical expenditures included all the expenditures of the treatment course of ASCT within 100 days.

### Characteristics of the study population

The potential confounders considered in this study include age, sex, comorbidities, including hypertension, diabetes mellitus, chronic obstructive pulmonary disease, coronary artery disease (CAD), heart failure, end-stage renal disease (ESRD), cerebrovascular accidents, liver cirrhosis, and autoimmune diseases, disease stage, and socioeconomic status. Patients’ socioeconomic status was categorized by degree of urbanization and level of monthly salary income stratified according to the previous work^18^.

### Statistical analysis

Patients’ demographic and clinical characteristics were presented as the total number (*n*) and proportion (%) for categorical data, and medians and interquartile ranges (IQR) for continuous data. Patients’ demographic data were compared by using the chi-square test for categorical variables, and the Mann–Whitney *U* test for continuous variables.

In the survival analysis, the Kaplan–Meier method was used for estimation of cumulative incidence of mortality, and differences between groups were tested using a log-rank test. Hazard ratios (HRs) and 95% confidence intervals (CIs) were calculated using Cox proportional hazards models, controlling for potential confounding factors in the multivariate model. All factors with *p* < 0.1 in the univariate analysis were included in the multivariate analysis. Sensitivity analyses were performed using different cutoffs (60 days and one year after ASCT) to evaluate the mortality risk between the HEPA and non-HEPA groups.

Furthermore, to eliminate bias in selection, propensity score matching at a 1:1 ratio using greedy matching techniques was performed to match the HEPA and non-HEPA groups. Propensity scores were calculated using age, sex, comorbidities, stage, degree of urbanization, income level, and medications in a logistic regression model. Data management and all statistical analysis were performed using SAS 9.4 software (SAS Institute Inc., Cary, NC, USA) and STATA statistical software, version 15.1 (StataCorp, College Station, TX, USA). All statistically significant levels were set at *p* < 0.05.

## Results

### Clinical characteristics of the study population

**Figure 1:**
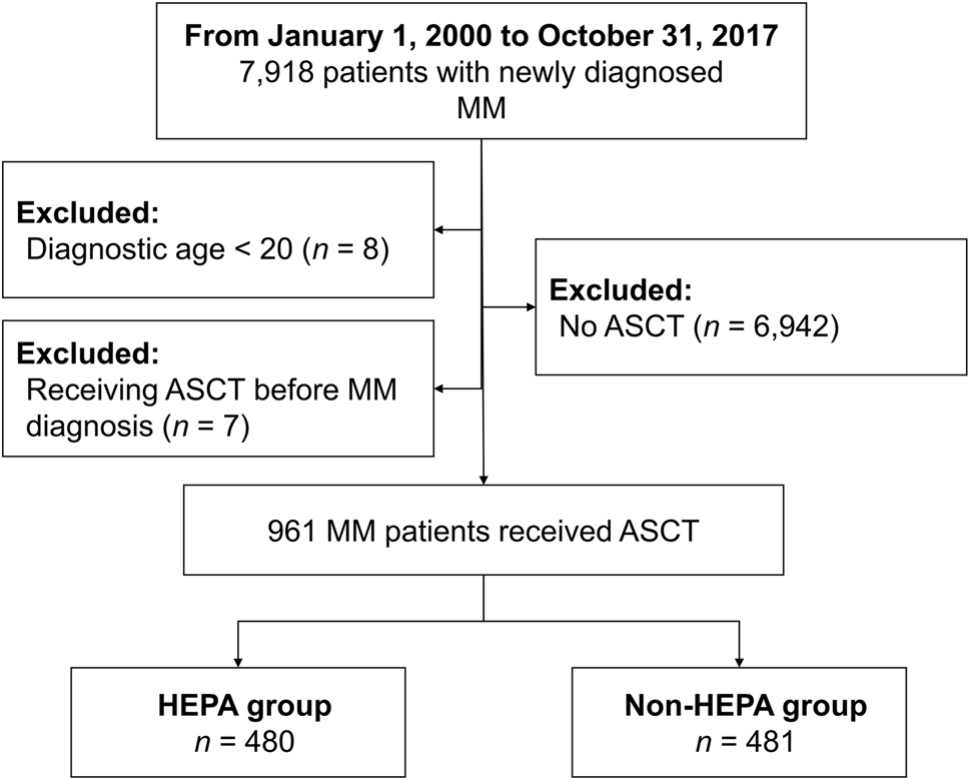
Patient selection flowchart.

We enrolled 7918 patients with newly diagnosed MM in Taiwan between January 1, 2000 and December 31, 2017. Patients under 20 years of age (*n* = 8), without ASCT (*n* = 6942), or receiving ASCT before MM (*n* = 7) were excluded. Finally, a total of 961 MM patients received ASCT during the 18-year study period (Fig. 1). The median age was 57 (range 28–76), and 54.9% were men. Hypertension (50.6%), diabetes mellitus (28.6%), chronic obstructive pulmonary disease (COPD) (27.1%), and CAD (25.8%) were the most common comorbidities. In regard to socioeconomic status, 59.4%, 33.4%, and 5.3% of the patients lived in urban, suburban, and rural areas, respectively. Of them, 480 patients (49.9%) received ASCT in the HEPA group. Age and having ESRD were different between the HEPA group and non-HEPA group. Patient characteristics are shown in Table 1.

**Table 1:** Baseline characteristics of patients receiving ASCT for multiple myeloma.

Characteristics	Total *n* = 961	HEPA group *n* = 480	Non-HEPA group *n* = 481	*p* value
Median age, years (range)	57 (28–76)	56 (28–73)	57 (28–76)	0.002
**Age, years**				
< 60	619 (64.4)	329 (68.5)	290 (60.3)	0.008
≥ 60	342 (35.6)	151 (31.5)	191 (39.7)	
**Sex**				
Male	528 (54.9)	276 (57.5)	252 (52.4)	0.111
Female	433 (45.1)	204 (42.5)	229 (47.6)	
Duration from MM to HSCT ≥ 180 days	667 (69.4)	341 (71.0)	326 (67.8)	0.272
**Comorbidities**				
Atrial fibrillation	21 (2.2)	10 (2.1)	11 (2.3)	0.829
Coronary artery disease	248 (25.8)	125 (26.0)	123 (25.6)	0.868
Liver cirrhosis	26 (2.7)	10 (2.1)	16 (3.3)	0.235
COPD	260 (27.1)	121 (25.2)	139 (28.9)	0.198
Cerebrovascular accidents	110 (11.4)	51 (10.6)	59 (12.3)	0.424
Diabetes mellitus	275 (28.6)	141 (29.4)	134 (27.9)	0.603
Hypertension	486 (50.6)	232 (48.3)	254 (52.8)	0.165
ESRD	150 (15.6)	63 (13.1)	87 (18.1)	0.034
Heart failure	93 (9.7)	47 (9.8)	46 (9.6)	0.905
Autoimmune disease	90 (9.4)	38 (7.9)	52 (10.8)	0.124
**Stage**				
I	122/477 (25.6)	49/193 (25.4)	73/282 (25.9)	0.531
II	159/477 (33.3)	70/193 (36.3)	89/282 (31.6)	
III	194/477 (41.1)	74/193 (38.3)	120/282 (42.6)	
**Degree of urbanization**				
Urban	571 (59.4)	288 (60.0)	283 (58.8)	0.899
Suburban	321 (33.4)	161 (33.5)	160 (33.3)	
Rural	51 (5.3)	24 (5.0)	27 (5.6)	
Unknown	18 (1.9)	7 (1.5)	11 (2.3)	
**Income level**				
Low	474 (49.3)	230 (47.9)	244 (50.7)	0.621
Intermediate	256 (26.6)	129 (26.9)	127 (26.4)	
High	231 (24.0)	121 (25.2)	110 (22.9)	

*ASCT* autologous stem cell transplantation; *IQR* interquartile range; *COPD* chronic obstructive pulmonary disease; *ESRD* end-stage renal disease.

### Treatment-related mortality rate

The 100-day mortality rate was 1.5% and 1.0% for the HEPA and non-HEPA groups, respectively. The crude hazard ratio (HR) for the 100-day mortality HEPA group was 1.39 (95% confidence interval [CI] 0.44–4.37, *p* = 0.576) compared to the non-HEPA group. In the univariate analysis, CAD (HR 5.80) and ESRD (HR 5.46) were associated with 100-day mortality. After adjusting for the variables found in univariate analysis, the 100-day mortality rate still had no difference between the HEPA and non-HEPA groups (adjusted HR 1.65, 95% CI 0.52–5.23, *p* = 0.399). Additionally, CAD and ESRD were significant risk factors of 100-day mortality in the multivariate analysis (Table 2).

**Table 2:** Risk factors for 100-day mortality in multiple myeloma patients receiving ASCT.

Predictive variables	Univariate analysis		Multivariate analysis^a^	
	HR (95% CI)	*P* value	HR (95% CI)	*P* value
HEPA group	1.39 (0.44–4.37)	0.576	1.65 (0.52–5.23)	0.399
Age ≥ 60 years	0.93 (0.28–3.08)	0.902		
Sex (male)	1.17 (0.37–3.69)	0.789		
Duration from MM to HSCT ≥ 180 days	0.88 (0.26–2.92)	0.832		
**Comorbidities**				
Atrial fibrillation	4.28 (0.55–33.14)	0.164		
Coronary artery disease	5.80 (1.75–19.27)	0.004	5.42 (1.63–18.02)	0.006
Liver cirrhosis	*			
COPD	0.89 (0.24–3.31)	0.868		
Cerebrovascular accident	1.54 (0.34–7.04)	0.576		
Diabetes mellitus	1.78 (0.57–5.61)	0.324		
Hypertension	1.97 (0.59–6.56)	0.266		
ESRD	5.46 (1.76–16.93)	0.003	5.33 (1.70–16.68)	0.004
Heart failure	1.85 (0.41–8.44)	0.428		
Ischemic stroke	1.11 (0.14–8.56)	0.924		
Autoimmune disease	1.96 (0.43–8.94)	0.385		
**Degree of urbanization**				
Urban	Reference			
Suburban	1.03 (0.30–3.52)	0.961		
Rural	1.59 (0.20–12.93)	0.664		
**Income level**				
Low	Reference			
Intermediate	*			
High	*			

*ASCT* autologous stem cell transplantation; *HR* hazard ratio; *CI* confidence interval; *COPD* chronic obstructive pulmonary disease; *ESRD* end-stage renal disease.

^a^All factors with *p* < 0.1 in the univariate analysis were included in the Cox multivariate analysis. *Do not converge

### Overall survival, time to transplantation, length of stay, emergency room visits, and readmission rate

**Figure 2:**
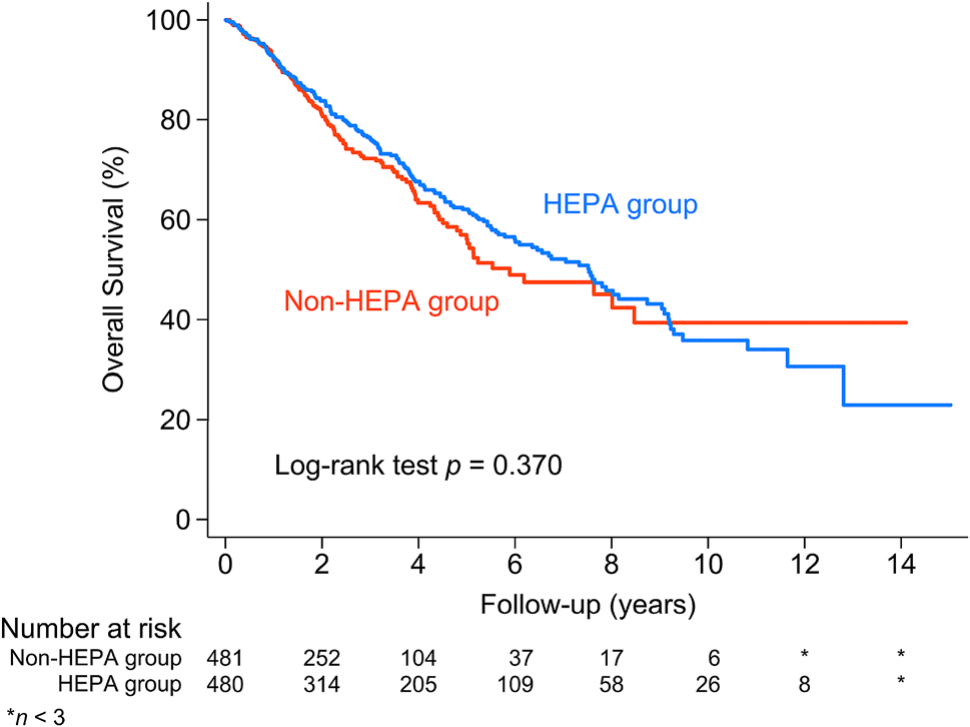
Overall survival of myeloma patients after autologous stem cell transplantation.

The median overall survival from ASCT was 7.52 years (95% CI 6.00–8.73) for the HEPA group, while it was 5.88 years (95% CI 4.99–8.46) for the non-HEPA group. There was no difference in the overall survival between the HEPA and non-HEPA group (*p* = 0.370, Fig. 2). The median time from MM diagnosis to ASCT was 7.5 (IQR 5.6–10.3) months and 7.0 (IQR 5.3–10.2) months for the HEPA and non-HEPA groups, respectively (*p* = 0.073). The median length of hospital stay for ASCT was longer for the HEPA group (24 [IQR 20–29] days for the HEPA group and 21 [IQR 18–26] days for the non-HEPA group, *p* < 0.001). There was no difference in emergency room visits within 14 days (8.3% and 6.4% for HEPA and non-HEPA, respectively) or the readmission rate within 14 days after discharge (6.5% and 4.2% for the HEPA and non-HEPA groups, respectively). The results of all the secondary endpoints are listed in Tables 3 and 4.

**Table 3:** Comparison of outcomes between the HEPA and non-HEPA groups.

Characteristics	Total *n* = 961	HEPA group^a^ *n* = 480	Non-HEPA group *n* = 481
100-day mortality	12 (1.2)	7 (1.5)	5 (1.0)
All-cause mortality	320 (33.3)	182 (37.9)	138 (28.7)
Median length of stay for treatment course of ASCT, days (IQR)	23 (19–28)	24 (20–29)	21 (18–26)
Emergency room visits within 14 days	71 (7.4)	40 (8.3)	31 (6.4)
Readmission within 14 days	51 (5.3)	31 (6.5)	20 (4.2)

*ASCT* autologous stem cell transplantation; *IQR* interquartile range.

^**a**^The HEPA group received ASCT in an isolation room with high-efficiency particulate air (HEPA) filtration.

**Table 4:** Comparison of outcomes between the HEPA and non-HEPA groups.

	Estimator	Univariate analysis		Multivariate analysis	
		Crude estimator (95% CI)	*P* value	Adjusted estimator^a^ (95% CI)	*P* value
100-day mortality	HR	1.39 (0.44–4.37)	0.576	1.65 (0.52–5.23)	0.399
All-cause mortality	HR	0.90 (0.72–1.13)	0.370	0.92 (0.74–1.16)	0.483
Length of stay for treatment course of ASCT ≥ median (23 days)	OR	1.97 (1.52–2.55)	< 0.001	1.50 (1.14–1.98)	0.004
Emergency room visits within 14 days	OR	1.32 (0.81–2.15)	0.264	1.39 (0.85–2.30)	0.194
Readmission within 14 days	OR	1.59 (0.89–2.83)	0.114	1.20 (0.64–2.25)	0.561

*OR* odds ratio; *HR* hazards ratio; *CI* confidence interval.

^a^Adjusted factors with *p* < 0.1 in the univariate analysis were included in the multivariate analysis.

### The healthcare cost

We further analyzed healthcare costs, including cost of hospitalization for ASCT and further treatment within 100 days, as well as outpatient services. The healthcare cost for ASCT inpatient care was $13,777.6 (IQR 11,772.1–16,696.7) and $6527.6 (IQR 4808.8–8601.4) for the HEPA and non-HEPA groups, respectively (*p* < 0.001). The cost of outpatient care was similar between the two groups (*p* = 0.249). Table 5 shows all the healthcare costs within 100 days of ASCT.

**Table 5:** Total expenditures among the HEPA and non-HEPA groups.

Characteristics	Total *n* = 961	HEPA group *n* = 480	Non-HEPA group *n* = 481	*p* value
**Median medical expenses, (IQR)**				
Outpatient costs within 100 days	1392.9 (689.8–2543.3)	1319.9 (698.5–2360.2)	1439.4 (684.2–2811.7)	0.249
Inpatient costs within 100 days	11,233.0 (6711.1–15,232.2)	14,409.8 (11,943.9–17,094.6)	6733.0 (4950.5–9256.8)	< 0.001

*IQR* interquartile range.

### Sensitivity analysis

We conducted sensitivity analyses using 60 days and one year as the cutoffs for transplant-related mortality (TRM). The 60-day mortality rate was 0.8% and 1.0% for the HEPA and non-HEPA groups (adjusted HR 0.92, 95% CI 0.25–3.47, *p* = 0.905) and the one-year mortality rate was 7.1% and 7.3% for the HEPA and non-HEPA groups (adjusted HR 1.00, 95% CI 0.62–1.61, *p* = 0.991), respectively. The results are consistent with our primary definition of TRM (100-day mortality; adjusted HR 1.65, 95% CI 0.52–5.23, *p* = 0.399).

### Propensity score–matched analysis

We conducted a propensity score***–***matched analysis to compare the HEPA and non-HEPA groups. We calculated the propensity scores for the likelihood of receiving ASCT in an isolation room with HEPA filtration using a multivariate logistic regression. We matched patients in the HEPA and non-HEPA groups with a 1:1 ratio (Supplemental Fig. S1). A total of 654 patients were matched. There was no statistically significant difference in the baseline characteristics between the two groups after matching (Supplemental Table S1). We further analyzed the primary endpoint and all secondary outcomes. There was also no statistically significant difference in the 100-day mortality rate (adjusted HR 1.83, 95% CI 0.54–6.27, *p* = 0.335) between the HEPA and non-HEPA groups. The 60-day, 1-year, and all-cause mortality rate, as well as emergency room visits and readmission within 14 days, were not statistically different (Supplemental Table S2). No overall survival difference between the HEPA and non-HEPA group were observed after matching (*p* = 0.089, Supplemental Fig. S2). However, the HEPA group had three more days of hospitalization and spent approximately $6500 more compared with the non-HEPA group (Supplemental Table S3).

## Discussion

To the best of our knowledge, this is the first population-based study that compares early mortality rates of MM patients receiving ASCT in an isolation room with HEPA filtration (the HEPA group) to those in a standard ward (the non-HEPA group). Our study reveals no difference in 100-day mortality, OS, emergency room visits, or readmission rate between the HEPA and non-HEPA groups receiving ASCT. However, the healthcare cost was higher for the HEPA group, and the time from diagnosis to ASCT was marginally increased (*p* = 0.073). Our results may help hematologists and healthcare administrators more appropriately allocate HSCT resources.

We have systematically reviewed existing studies regarding ASCT in MM patients. We found many MM patients receiving ASCT in an isolation room with HEPA filtration^8,9,19,20^. Our study also reveals that half of the ASCT in MM patients was performed in an HSCT special ward with such facilities, which implies that many hematologists still believe that an isolation room with HEPA filtration can reduce infection and early-mortality rates in those patients. Our study discloses a very low mortality rate for MM patients receiving ASCT whether in an isolation room with HEPA filtration or not. The effect of HEPA filtration was to lower the nosocomial invasive fungal infection rate^12^. However, our previous study indicated low incidence of invasive fungal infection in MM patients^15^. Therefore, the insignificance of treatment-related mortality between the HEPA and non-HEPA group might be related to the low incidence of invasive fungal infection in MM patients.

In addition, we found that CAD and ESRD were independent risk factors of TRM among MM patients receiving ASCT. Tsakiris et al. reported that the median OS of MM patients receiving renal replacement therapy was only 0.91 years^21^. Lee et al*.* reported that MM patients with dialysis-dependent renal failure had a median survival of 3.4 years from ASCT, and 22.0% of the patients became dialysis independent after ASCT^22^. The 60-day, 100-day, and 1-year mortality rates of the ESRD patients in our cohort were 2.7%, 4.0%, and 8.7%, respectively.

ASCT should be performed with caution in MM patients with CAD. Stillwell et al. reported that TRM and one-year mortality were 5.6% and 15.3% in CAD patients receiving HSCT. Among them, 68.1% received ASCT^23^. Our study reveals that the 100-day mortality rate of CAD patients receiving ASCT was 3.2%, which was much higher than those without CAD. Saad et al. analyzed the records in the Center for International Blood and Marrow Transplant Research database and concluded that hematopoietic cell transplant comorbidity index (HCT-CI), including CAD and renal dysfunction, could predict survival in MM patients receiving ASCT^24^.

We found that the HEPA group had three more days of hospitalization and spent $7250 more for ASCT, in comparison with the non-HEPA group. However, all the short-term outcomes and long-term survival of the two groups had no difference. This population-based study shows that an isolation room with HEPA might not be necessary for MM patients receiving ASCT. Furthermore, ambulatory HSCT or even at-home HSCT, emphasizing on outpatient visits only or early discharge after stem cell transfusion, had been demonstrated to be feasible, safe, and cost-effective in several studies, especially for carefully selected MM patients undergoing ASCT^25–27^. A meta-analysis even showed a lower chance of febrile neutropenia and septicemia with outpatient ASCT^28^. The necessity of HEPA filtration for MM patients receiving ASCT should be re-examined.

Some studies have different definitions of early mortality and TRM in MM. Reece reported 100-day and one-year TRM mortality for patients receiving ASCT. Schmidt-Hieber, Kumar, Yin reported 100-day and one-year TRM for allogeneic transplantation^32–35^. Schmidt-Hieber, Kröger, Kuruvilla used one year as the cutoff for TRM for patients receiving allogeneic transplantation^29–31^. Bringhen, Augustson, Larocca used 60 days as the cutoff for early mortality in MM studies^36–38^. We conducted sensitivity analysis showing that neither 60-day, 100-day, nor one-year mortality had any statistical difference between HEPA and non-HEPA groups. These results consistently demonstrate that ASCT in MM is fairly safe whether the patients are treated in an isolation room with HEPA or not.

The non-HEPA group was older and had a higher proportion of ESRD. We conducted a propensity score–matched analysis. After matching, all the patient characteristics were similar. Both groups still had no difference in 60-day, 100-day, and one-year mortality, OS, emergency room visits, or readmission within 14 days. The HEPA group nevertheless had a higher medical expenditure and longer length of stay after matching. Saini et al. reported MM patients with t(11;14) had similar outcomes as those with normal cytogenetic and FISH studies in a propensity score–matched analysis^39^. Varma et al. reported that MM patients with 1q + /1p–were at significantly increased risk of progression or death compared to the propensity score–matched comparison group^40^. In the present study, the propensity score was defined as the conditional probability of receiving ASCT in an isolation room with HEPA. The calculated score was used to balance the covariates in the two groups and therefore reduced the bias^41^.

Our study has some limitations. Most of our patients didn’t receive genetic studies by FISH, which are required according to the Revised International Staging System^42^. Second, this study has inherent limitations by using administrative data that did not provide smoking status, performance status, disease status prior to ASCT, comprehensive medications, and some essential laboratory data. Finally, the HEPA and non-HEPA groups were not randomly assigned, so confounding factors might exist. However, there was no survival difference between the two groups, although the non-HEPA group was slightly older and more of them had ESRD.

In conclusion, some hematologists believe an isolation room with HEPA filtration can reduce complications of hematologic patients receiving ASCT. We found that about 50% of the MM patients in Taiwan received ASCT in an isolation room with HEPA filtration, and it didn’t affect 100-day mortality. This study may help clinicians and healthcare administrators utilize the limited resources of HSCT facilities. Further validation of our findings in other cohorts is warranted.

## Supplementary Information

Supplementary Information.
