# Supplementary material for: The influence of high-efficiency particulate air filtration on mortality among multiple myeloma patients receiving autologous stem cell transplantation
Source: Sci Rep. 2021 Jun 3;11:11789. doi: 10.1038/s41598-021-91135-0 (PMC8175695; doi:10.1038/s41598-021-91135-0)

**TITLE PAGE**

**Full Title:**

The Influence of High-Efficiency Particulate Air Filtration on Mortality Among Multiple Myeloma Patients Receiving Autologous Stem Cell Transplantation

**Running Title:**

Role of HEPA Filtration in Myeloma Patients Receiving ASCT

**Authors:**

Chun-Kuang Tsai, MD^1^, Chiu-Mei Yeh, MS^1,2^, Ying-Chung Hong, MD^3^, Po-Min Chen, MD, PhD^1,4^, Jin-Hwang Liu, MD, PhD^5,6^, Jyh-Pyng Gau, MD^1,4^, Chia-Jen Liu, MD, PhD^1,2,4§^

**Author Affiliations:**

^1^ Division of Hematology and Oncology, Department of Medicine, Taipei Veterans General Hospital, Taipei, Taiwan

^2^ Institute of Public Health, National Yang-Ming University, Taipei, Taiwan

^3^ Division of Hematology and Oncology, Kaohsiung Veterans General Hospital, Kaohsiung, Taiwan

^4^ School of Medicine, National Yang-Ming University, Taipei, Taiwan

^5^ Division of Hematology and Oncology, Cheng Hsin General Hospital, Taipei, Taiwan

^6^ Chong Hin Loon Cancer and Biotherapy Research Center, and Institute of Biopharmaceutical Sciences, National Yang-Ming University, Taipei, Taiwan

**§Corresponding author:**

Chia-Jen Liu, MD, PhD

Division of Hematology and Oncology

Department of Medicine

Taipei Veterans General Hospital

No. 201 Shipai Road, Sec. 2

Taipei, Taiwan 11217

Tel: +886-2-28757529

Fax: +886-2-28757762

Email: [chiajenliu@gmail.com](mailto:chiajenliu@gmail.com)

**Email addresses:**

C-KT: jvictsai@gmail.com

C-MY: [s9311124@cycu.org.tw](mailto:s9311124@cycu.org.tw)

Y-CH: m8201136@gmail.com

P-MC: pmchen@vghtpe.gov.tw

J-HL: jhliu@vghtpe.gov.tw

J-PG: jpgau@vghtpe.gov.tw

C-JL: chiajenliu@gmail.com

**Supplemental Tables and Figures**

**Supplemental Table 1 Baseline characteristics of MM patients receiving ASCT after propensity score matching**

| **Characteristics** | **Total**  ***n* = 654** | **HEPA group**  ***n* = 327** | **Non-HEPA group**  ***n* = 327** | ***p* value** |
| --- | --- | --- | --- | --- |
| Median age, years (range) | 57 (28–74) | 57 (31–73) | 57 (28–74) | 0.863 |
| Age, years |  |  |  |  |
| < 60 | 417 (63.8) | 208 (63.6) | 209 (63.9) | 0.935 |
| ≥ 60 | 237 (36.2) | 119 (36.4) | 118 (36.1) |  |
| Sex |  |  |  |  |
| Male | 357 (54.6) | 178 (54.4) | 179 (54.7) | 0.937 |
| Female | 297 (45.4) | 149 (45.6) | 148 (45.3) |  |
| Duration from MM to ASCT ≥ 180 days | 465 (71.1) | 236 (72.2) | 229 (70.0) | 0.546 |
| Comorbidities |  |  |  |  |
| Atrial fibrillation | 17 (2.6) | 9 (2.8) | 8 (2.4) | 0.806 |
| Coronary artery disease | 169 (25.8) | 83 (25.4) | 86 (26.3) | 0.789 |
| Liver cirrhosis | 15 (2.3) | 7 (2.1) | 8 (2.4) | 0.794 |
| COPD | 172 (26.3) | 86 (26.3) | 86 (26.3) | 1.000 |
| Cerebrovascular accidents | 84 (12.8) | 37 (11.3) | 47 (14.4) | 0.243 |
| Diabetes mellitus | 183 (28.0) | 92 (28.1) | 91 (27.8) | 0.931 |
| Hypertension | 330 (50.5) | 157 (48.0) | 173 (52.9) | 0.211 |
| ESRD | 96 (14.7) | 46 (14.1) | 50 (15.3) | 0.659 |
| Heart failure | 64 (9.8) | 29 (8.9) | 35 (10.7) | 0.430 |
| Autoimmune disease | 70 (10.7) | 33 (10.1) | 37 (11.3) | 0.613 |
| Stage |  |  |  |  |
| I | 83/346 (24.0) | 42/175 (24.0) | 41/171 (24.0) | 0.811 |
| II | 134/346 (38.7) | 64/175 (36.6) | 70/171 (40.9) |  |
| III | 129/346 (37.3) | 69/175 (39.4) | 60/171 (35.1) |  |
| Degree of urbanization |  |  |  |  |
| Urban | 394 (60.2) | 189 (57.8) | 205 (62.7) | 0.539 |
| Suburban | 217 (33.2) | 113 (34.6) | 104 (31.8) |  |
| Rural | 31 (4.7) | 18 (5.5) | 13 (4.0) |  |
| Unknown | 12 (1.8) | 7 (2.1) | 5 (1.5) |  |
| Income level |  |  |  |  |
| < 20,000 | 314 (48.0) | 154 (47.1) | 160 (48.9) | 0.870 |
| 20,000–39,999 | 183 (28.0) | 92 (28.1) | 91 (27.8) |  |
| ≥ 40,000 | 157 (24.0) | 81 (24.8) | 76 (23.2) |  |
| Treatment |  |  |  |  |
| Bortezomib | 500 (76.5) | 254 (77.7) | 246 (75.2) | 0.461 |
| Melphalan | 50 (7.6) | 24 (7.3) | 26 (8.0) | 0.769 |
| Thalidomide | 540 (82.6) | 271 (82.9) | 269 (82.3) | 0.837 |
| Lenalidomide | 17 (2.6) | 8 (2.4) | 9 (2.8) | 0.806 |
| Cyclophosphamide | 595 (91.0) | 297 (90.8) | 298 (91.1) | 0.891 |
| Doxorubicin | 83 (12.7) | 43 (13.1) | 40 (12.2) | 0.725 |

ASCT, autologous stem cell transplantation; IQR, interquartile range; COPD, chronic obstructive pulmonary disease; ESRD, end-stage renal disease; MM, multiple myeloma

**Supplemental Table 2 The association between HEPA filtration and outcomes after propensity score matching**

|  |  | **Univariate analysis** | |  | **Multivariate analysis** | |
| --- | --- | --- | --- | --- | --- | --- |
|  | **Estimator** | **Crude estimator**  **(95% CI)** | ***P* value** |  | **Adjusted estimator**^a^  **(95% CI)** | ***P* value** |
| Mortality | HR |  |  |  |  |  |
| 60-day mortality | HR | 0.99 (0.25–3.96) | 0.990 |  | 1.08 (0.27–4.34) | 0.913 |
| 100-day mortality | HR | 1.73 (0.51–5.92) | 0.381 |  | 1.83 (0.54–6.27) | 0.335 |
| 1-year mortality | HR | 0.83 (0.49–1.40) | 0.476 |  | 0.82 (0.48–1.39) | 0.458 |
| All-cause mortality | HR | 0.82 (0.63–1.06) | 0.128 |  | 0.81 (0.63–1.05) | 0.115 |
| Length of stay for treatment course of ASCT ≥ median (23 days) | OR | 1.64 (1.20–2.24) | 0.002 |  | 1.72 (1.25–2.36) | 0.001 |
| Emergency room visits within 14 days | OR | 0.78 (0.42–1.45) | 0.431 |  | 0.75 (0.40–1.42) | 0.383 |
| Readmission within 14 days | OR | 0.69 (0.33–1.48) | 0.345 |  | 0.69 (0.32–1.49) | 0.340 |

OR, odds ratio; HEPA, high-efficiency particulate air; HR, hazards ratio; CI, confidence interval

^a^Adjusted factors with *p* < 0.1 in the univariate analysis were included in the multivariate analysis.

**Supplemental Table 3 Total expenditures of ASCT among the HEPA and non-HEPA groups after propensity score matching**

| **Characteristics** | **Total**  ***n* = 654** | **HEPA group**  ***n* = 327** | **Non-HEPA group**  ***n* = 327** | ***p* value** |
| --- | --- | --- | --- | --- |
| Median medical expenses, (IQR) |  |  |  |  |
| Outpatient costs within 100 days | 1,478.8  (727.6–2,666.5) | 1,530.3  (803.0–2,726.8) | 1,408.9  (657.9–2,543.3) | 0.304 |
| Inpatient costs within 100 days | 11,018.9  (6,898.3–14,493.6) | 13,373.4  (11,406.6–15,994.8) | 6,945.9  (4,967.6–10,097.5) | < 0.001 |

ASCT, autologous stem cell transplantation; IQR, interquartile range

**Supplemental Figure 1 The propensity score distribution among the HEPA and non-HEPA groups before and after matching**

**
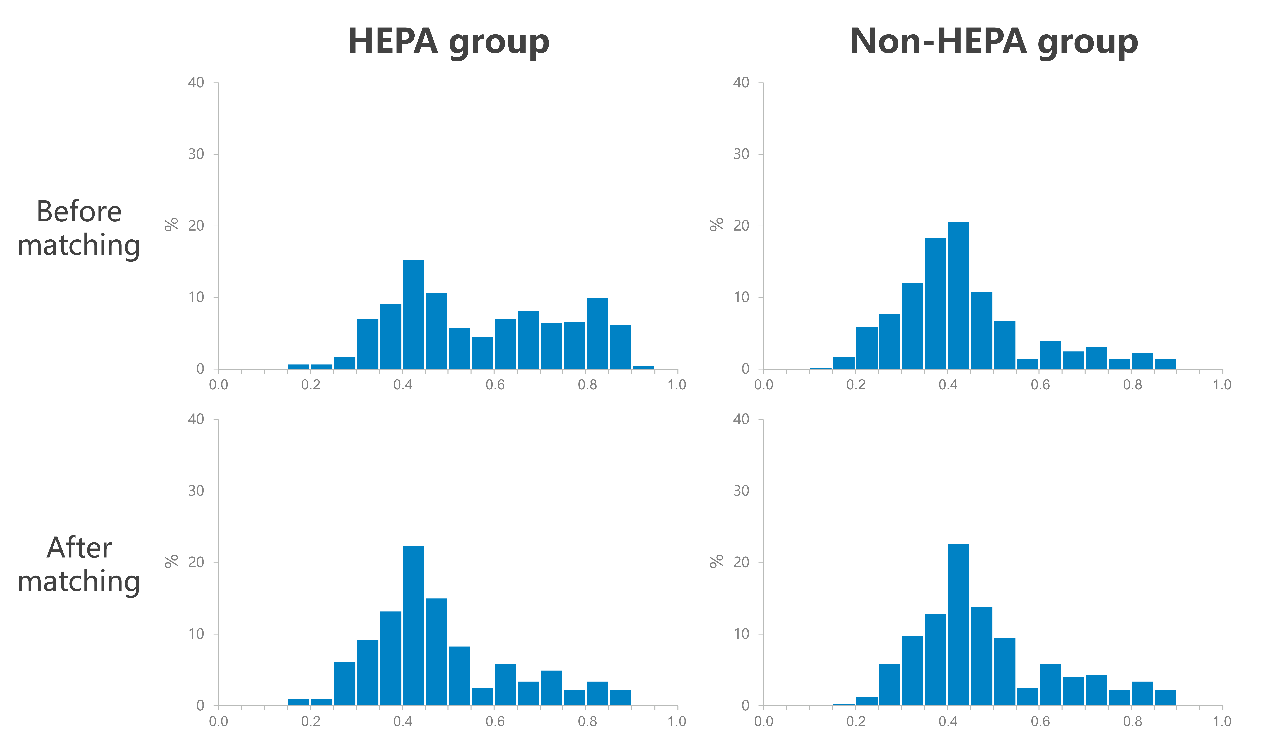
**

**Supplemental Figure 2 Overall survival among the HEPA and non-HEPA groups after propensity score matching**


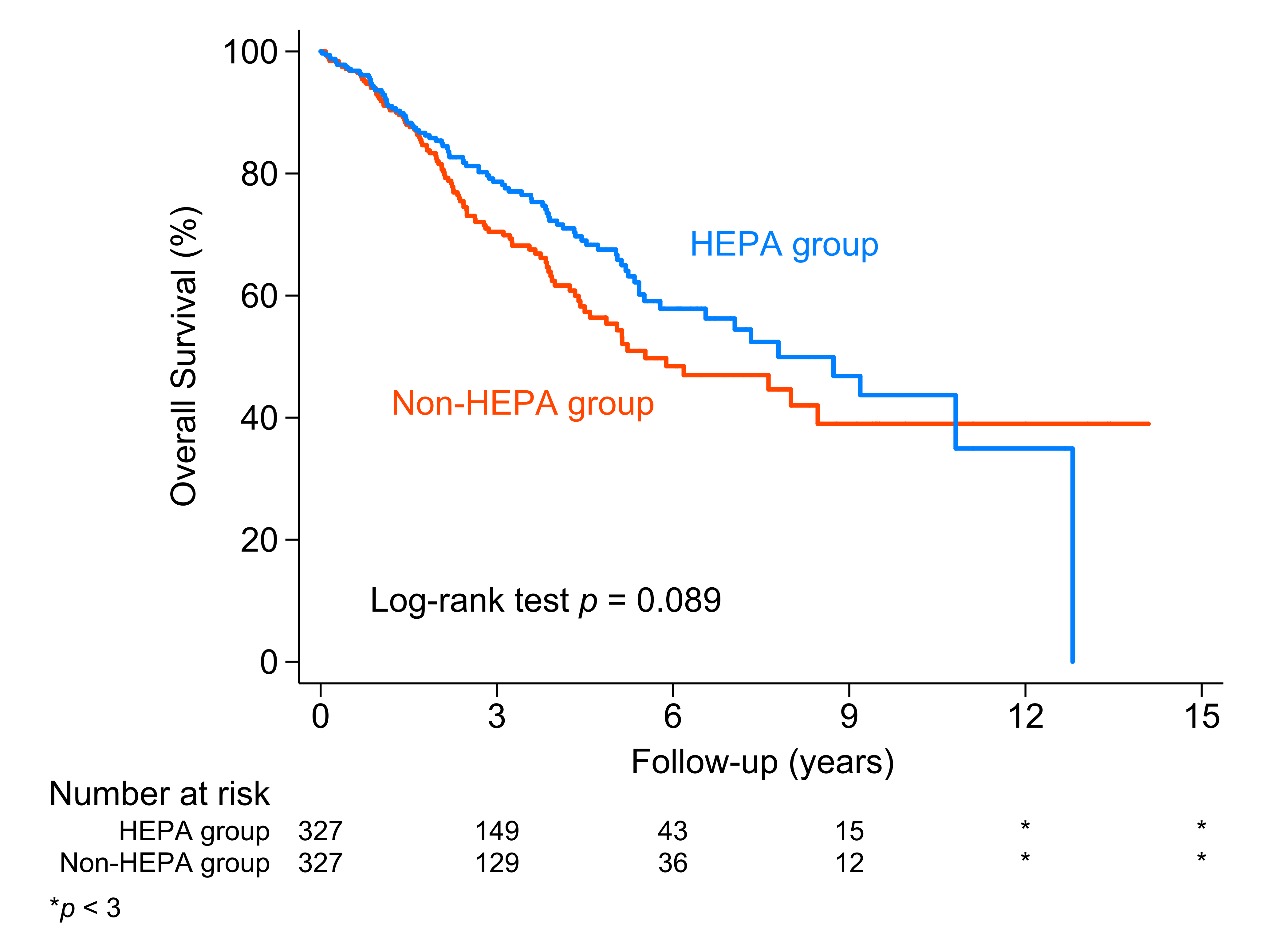

Supplement: Supplementary file 1 — Supplementary Information. [file 41598_2021_91135_MOESM1_ESM.docx]
